# Video consultation in general practice during COVID-19: a register-based study in Denmark

**DOI:** 10.3399/BJGPO.2023.0208

**Published:** 2024-04-17

**Authors:** Ulrik Bak Kirk, Claus Høstrup Vestergaard, Bodil Hammer Bech, Morten Bondo Christensen, Per Kallestrup, Linda Huibers

**Affiliations:** 1 Department of Public Health, Aarhus University, Aarhus, Denmark; 2 Research Unit for General Practice, Aarhus, Denmark

**Keywords:** telemedicine, COVID-19, general practitioners, primary healthcare, video consultations, remote consultation

## Abstract

**Background:**

During the COVID-19 pandemic, general practices in Denmark rapidly introduced video consultations (VCs) to prevent viral transmission.

**Aim:**

To study the use of VCs in daytime general practice by describing the rate of VCs, and the patient characteristics associated with having VCs.

**Design & setting:**

Register-based study of consultations in daytime general practice in Denmark.

**Method:**

We included all consultations in daytime general practice from 1 January 2019–30 November 2021. We calculated the rate of video use and categorised the general practices into no, low, and high use. Logistic regression was used to calculate adjusted odds ratios (aOR) for having a VC for different patient characteristics when contacting a video-using practice, stratified for low- and high-using practices.

**Results:**

A total of 30 148 478 eligible consultations were conducted during the pandemic period. VCs were used mostly during the early stage pandemic period, declining to about 2% of all clinic consultations in the late-stage period. Patients having more VCs were young, had a long education, were employed, and lived in big cities. In low-using practices, native Danes and 'western' immigrants had higher odds of receiving a VC than 'non-western' immigrants, and patients with ≥2 comorbidities had lower odds than those without comorbidities.

**Conclusion:**

Patients of a younger age, with long education, or employment had higher odds of receiving a VC, while patients of an older age and patients who had retired had lower odds. This difference in the access to VCs warrants further attention.

## How this fits in

Governments upscaled remote care during the early stage COVID-19 pandemic period, and video consultations (VCs) were rapidly introduced in general practices in Denmark to prevent viral transmission. This study’s aim was to investigate the use of VCs in daytime general practice by describing the rate of VCs, and the patient characteristics associated with having VCs. Patients of a younger age, with long education, and current employment had higher odds of a VC, while patients of an older age and patients who had retired had lower odds. This difference in the access to VCs warrants further attention, and more insight is needed for GPs to sustainably deliver VCs with the right patient at the right time, optimising future use of VCs.

## Introduction

The World Health Organization formally declared the COVID-19 outbreak a pandemic on 11 March 2020.^1^ As primary care is the entry point into the health system,^2^ GPs have been pivotal to the pandemic response as the first point of contact for possibly infected patients, as gatekeepers to the healthcare system, as coordinators of care, and as organisers of follow-up care for most patients.^3,4^

During the pandemic, the GPs had to balance between managing the risk of transmission and providing regular care.^5^ To prevent transmission, GPs started to use more existing remote consultation services,^6^ such as email and telephone consultations, but they also rapidly introduced a new modality in the shape of video consultations (VCs) wherever possible.^7^ A VC can be defined as a '*remote, synchronous consultation type which facilitates audio-visual two-way communication between GP and patient and adds body language and gestures to the GP–patient interactio*n'.^8^

Numerous studies have shown VCs to be acceptable, safe, and effective.^9,10^ However, the use of VCs has previously been examined in selected patient groups and often in intervention studies rather than in a real-world setting.^11^ VCs may help maintain access to primary care, yet remote consultations may concurrently exacerbate existing differences in the access to health care.^12,13^ Governments upscaled remote care during the COVID-19 pandemic.^14^ However, more insight is needed for GPs to sustainably deliver VCs with the right patient at the right time in general practice. Therefore, we aimed to study the use of VCs in daytime general practice during the COVID-19 pandemic by describing the rate of video-using GP practices, and the patient characteristics associated with utilising VCs.

## Method

### Design and population

We conducted a cross-sectional register-based study, including all clinic consultations with Danish daytime general practice from 1 January 2019– 30 November 2021.

### Setting

In Denmark, general practice is tax-funded and free of charge for the patient. During the day (8 am–4 pm), GPs provide care to their listed patients and offer a range of basic services, including face-to-face clinic consultations, telephone and email consultations, and home visits. The remuneration of GPs is based on a mix of fee-for-services (70–75%) and capitation (25–30%).^15^ Thus, GPs are economically incentivised to code their services correctly and completely.

From 1 April 2020, VCs were available through the My Doctor app, which was recommended by the Organisation of General Practitioners in Denmark,^16^ and both the GP practice and the patient could suggest video use. In addition to VCs, the option of a so-called extended telephone consultation was introduced, and new (temporary) remuneration codes for reimbursement were presented (Supplementary Table S1). A video remuneration code was available pre-pandemic owing to a pilot VC project, with very low activity.

### Data collection

We collected data from a range of national registers, using the personal identification number to link registers. The National Health Insurance Service Register^17^ provided information on date, time, and type of contact with daytime GP (through remuneration codes). The National Patient Register^18^ provided the diagnosis codes needed to calculate the level of comorbidity (that is, the number of diagnoses included in the Charlson Comorbidity Index). The Danish Civil Registration System^19^ and Statistics Denmark delivered data on patient characteristics (that is, age, sex, cohabitation, income, urbanisation, employment status, education, and ethnic group). Apart from age, sex, and comorbidity, all covariates were estimated at household level to avoid excluding contacts for children because of missing values.

Clinic consultations were defined as face-to-face clinic consultations, clinic consultations for chronic care, and clinic consultations for conversational therapy. Consequently, these clinic consultations were considered VCs when coded as video with the temporary remuneration codes.

### Data handling

General practices were included if they were active during the study period. This was defined as having ≥300 clinic consultations during the early stage pandemic period (15 March 2020–13 June 2020) and ≥700 clinic consultations during the late-stage pandemic period (14 June 2020–30 November 2021). This approach was taken to ensure valid calculation of the rate of video use.

Next, we categorised covariates (Supplementary Table S2) into age groups (0–10, 11–20, 21–30, 31–40, 41–50, 51–60, 61–70, 71–80, 81–90, >90 years), cohabitation (single, cohabiting, married), years of education (<10, 10–15, >15 years), ethnic group (native born, western immigrant, non-western immigrant), comorbidity level (0, 1, 2, ≥3 diseases), income (in quintiles), urbanisation level (>100 000, 20 000–100 000, 1000–19 999, <1000 inhabitants), employment status (employed, unemployed, retired), and administrative region (Capital Region of Denmark, North Denmark Region, Central Denmark Region, Region of Southern Denmark, Region Zealand).

Information on sex, age, and comorbidity was complete, but missing values were present for socioeconomic covariates. As this information was often missing concurrently and caused convergence issues for the model, we excluded all contacts with missing values (*n* = 568 629; 1.8%).

### Analyses

**Figure 1.**
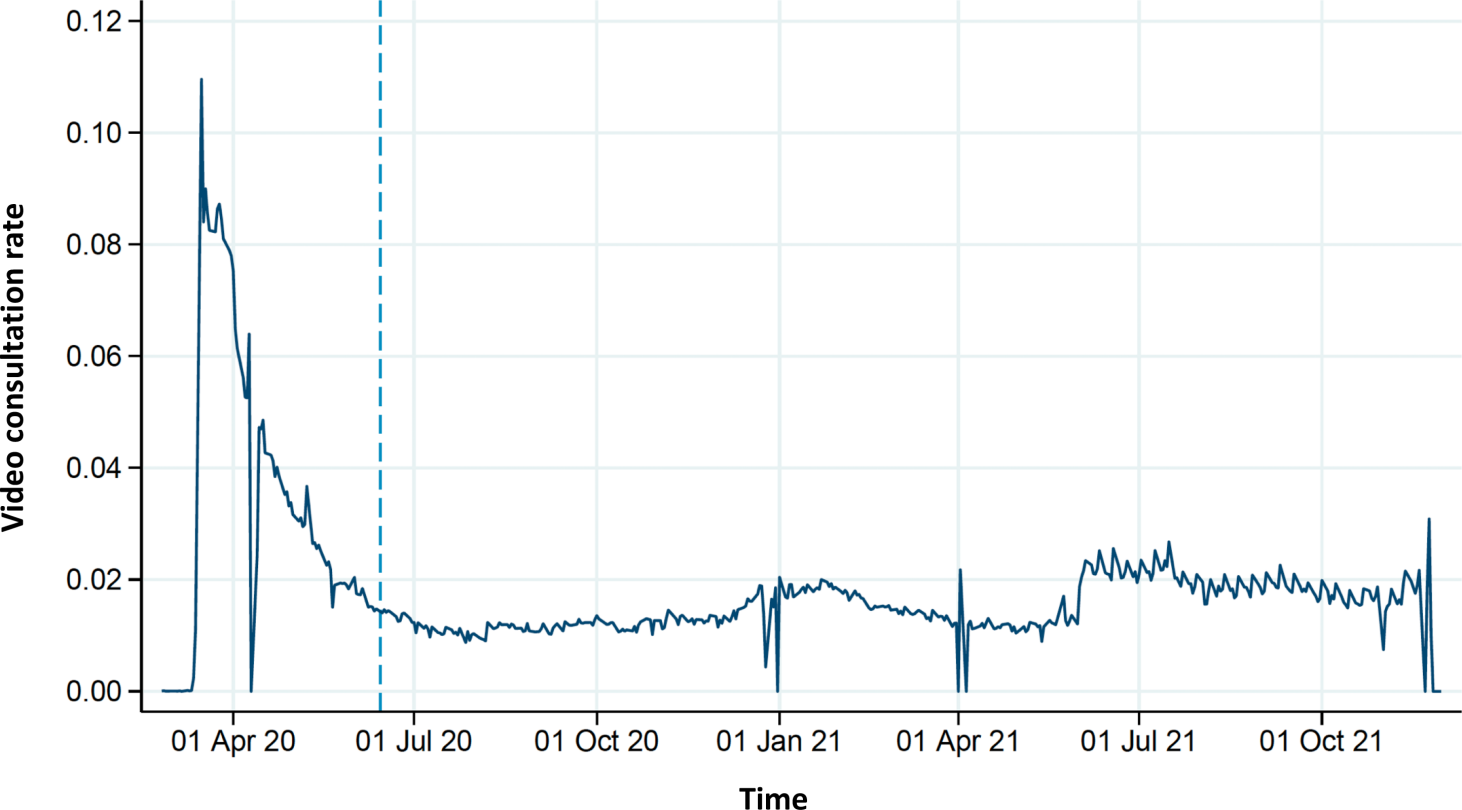
Video consultation rate over time for all clinic consultations (end of early stage pandemic period indicated). Pandemic period: 15 March 2020–30 November 2021. Early stage pandemic period: 15 March 2020–13 June 2020. Late-stage pandemic period: 14 June 2020–30 November 2021. Clinic consultations included regular (face-to-face) clinic consultations, video consultations, chronic care, and conversational therapy; weekends and public holidays were excluded.

We calculated the rate of VCs per 100 clinic consultations in general practice for the entire pandemic period to take into account any user variations (Figure 1). Based on this, we split the data into an early stage period (15 March 2020–13 June 2020) and a late-stage period (14 June 2020–30 November 2021). We also calculated the rates of VCs in the practice based on both the early and late-stage periods and categorised general practices into no use (rate <1%), low use (rate 1–3%), and high use (rate >3%). These cut-offs were based on existing evidence on video use,^20–23^ data from Denmark, and clinical experience. A sensitivity analysis showed similar results when a cut-off point of five VCs per 100 contacts was used to define high use.

To describe the patient populations before the rapid implementation of VCs owing to COVID-19, we used pre-pandemic data (1 January 2019–14 March 2020) to present the patient population receiving face-to-face clinic consultations in general practice. These data were stratified for the level of video use in the GP practices, and calculations were based on the early and late-stage pandemic periods (Table 1).

**Table 1.** Description of patient characteristics of pre-pandemic practice populations,^a^ according to level of video use in GP practices during the early stage and the late-stage pandemic periods, respectively (in percentages)

	Patient population in pre-pandemic period (*n* = 21 640 831)					
	**Level of video use based on early stage pandemic period** ^b^			**Level of video use based on late-stage pandemic period** ^c^		
**Characteristics**	**No use** ^d^	**Low use** ^e^	**High use** ^f^	**No use** ^d^	**Low use** ^e^	**High use** ^f^
***N***	4 869 114	6 727 198	10 044 519	14 088 199	4 690 997	2 861 635
(%)	22.5	31.1	46.4	65.1	21.7	13.2
**Sex**						
Female	57.4	58.9	60.1	58.4	60.1	61.2
Male	42.6	41.1	39.9	41.6	39.9	38.8
**Age group, years**						
0–10	6.5	6.9	7.6	6.7	7.7	8.3
11–20	7.3	7.6	7.6	7.6	7.8	7.0
21–30	9.9	10.4	12.9	9.9	12.2	17.7
31–40	9.7	10.1	11.4	9.8	11.3	13.5
41–50	11.8	12.0	12.2	11.9	12.3	12.1
51–60	14.7	14.6	14.0	14.7	14.1	13.1
61–70	15.0	14.4	13.3	14.7	13.4	11.6
71–80	16.3	15.5	13.6	16.0	13.8	10.9
81–90	7.7	7.4	6.4	7.6	6.4	5.0
>90	1.2	1.1	1.0	1.1	1.0	0.8
**Cohabitation**						
Single	33.5	31.9	32.9	32.2	32.2	35.9
Cohabiting unmarried, or widowed, or divorced	13.7	14.4	15.6	14.0	15.5	17.4
Married	52.8	53.7	51.5	53.8	52.2	46.7
**Educational level, years**						
<10	20.8	18.5	16.7	19.2	16.8	15.4
10–15	48.9	48.9	46.7	48.9	47.2	44.1
>15	30.4	32.6	36.7	32.0	35.9	40.6
**Ethnic group**						
Non-western immigrants	8.2	6.2	7.2	6.6	7.1	9.9
Western	2.5	2.1	2.3	2.2	2.2	2.8
Native Danish	89.3	91.7	90.5	91.2	90.8	87.3
**Comorbidity** (*n*)						
None	74.3	75.4	77.7	75.0	77.4	80.5
1	18.1	17.6	16.3	17.8	16.4	14.5
2	5.3	5.0	4.3	5.1	4.4	3.6
≥3	2.3	2.1	1.7	2.1	1.8	1.4
**Income^a^** (quintiles)						
first	14.9	13.0	14.8	13.4	14.1	18.6
second	22.5	20.8	19.4	21.3	19.5	18.5
third	21.9	21.8	21.0	21.9	21.4	19.6
fourth	21.1	22.6	22.2	22.2	22.5	20.9
fifth	19.4	21.5	22.4	21.1	22.2	22.2
Negative or zero	0.2	0.2	0.2	0.2	0.2	0.3
**Urbanisation**						
>100 k	26.1	23.6	35.5	23.6	33.2	54.1
20 k–100 k	20.7	21.2	19.9	20.5	22.5	16.8
1 k–20 k	31.2	35.0	27.7	34.6	27.1	17.8
<1 k	22.0	20.1	16.9	21.3	17.2	11.3
**Employment status**						
Unemployed	9.9	8.6	8.7	8.9	8.7	9.6
Retired	29.2	27.9	24.4	28.6	24.7	19.7
Employed	60.8	63.6	66.8	62.5	66.6	70.7

^a^Pre-pandemic patient populations (1 January 2019–14 March 2020) for the three groups of video-using practices.^b^Video use calculated based on the early stage pandemic period (15 March 2020–13 June 2020).^c^Video use based on late-stage pandemic period (14 June 2020–30 November 2021). User rate based on entire pandemic period: ^d^No use: user rate <1%; ^e^Low use: user rate 1–3%; ^f^High use: user rate >3%.

**Figure 2.**
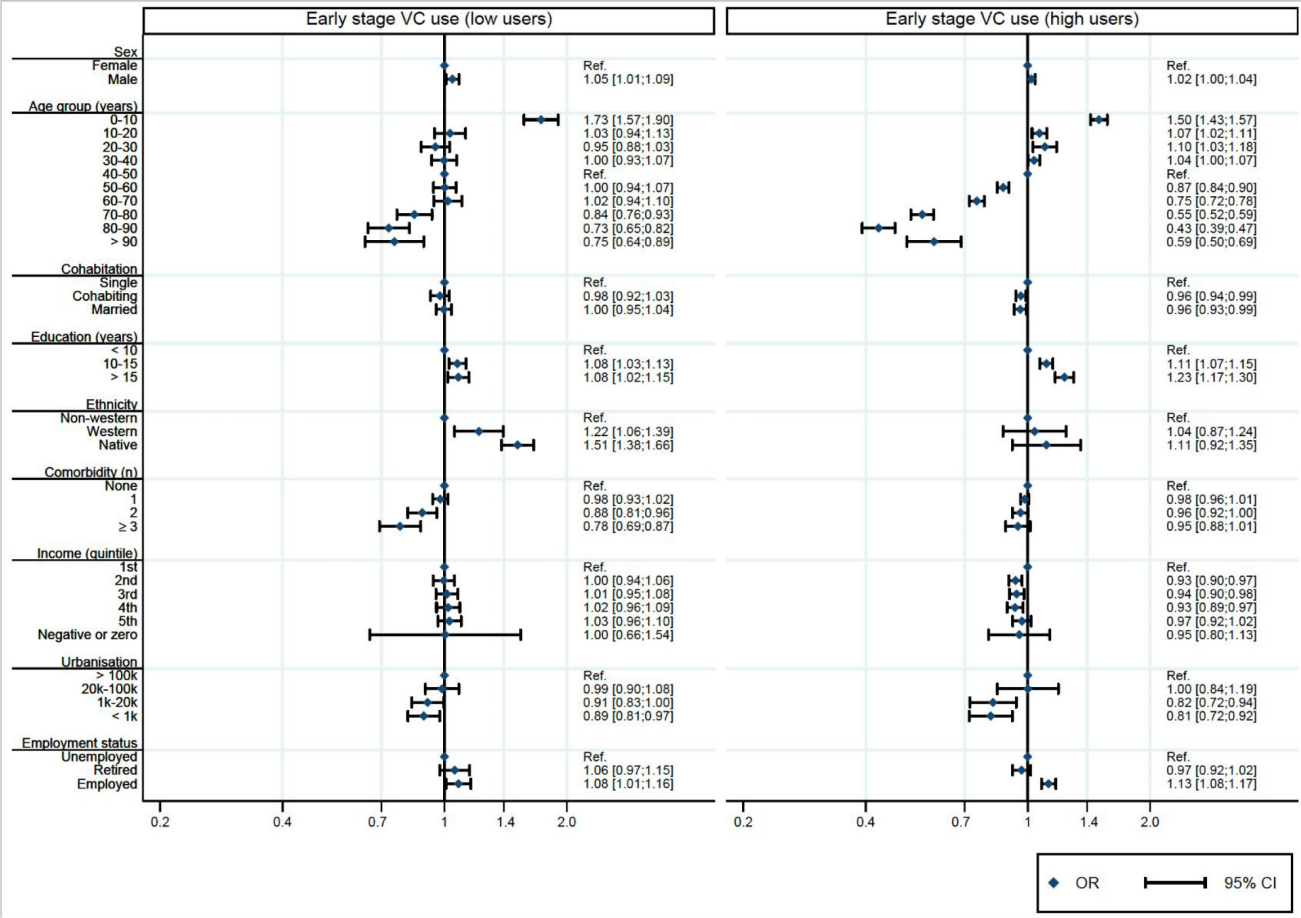
Adjusted^a^ odds ratio (95% confidence interval) for having a video consultation for different patient characteristics when contacting a video-using practice, stratified for low and high-using practices in the early stage pandemic. Definition of video-using GP practice based on early stage pandemic period (15 March 2020–13 June 2020). ^a^Mutually adjusted for variables included in the figure. 95% CI = 95% confidence intervals; OR = odds ratio. VC = video consultation.

To study patient characteristics associated with utilising VCs, we included patient consultations in video-using GP practices (that is, low use and high use). Next, we conducted multivariable logistic regression analyses to calculate the association between patient characteristics and VC performance (adjusted odds ratios [aOR], 95% confidence intervals [95% CI]). The following variables were included: sex, age, cohabitation, education level, ethnic group, comorbidity, income, urbanisation, and employment status. Two regression models were calculated: one for the early stage, and one for the late-stage pandemic period. Results were presented as forest plots (Figures 2 3). Stata (version 17.0) was used.

**Figure 3.**
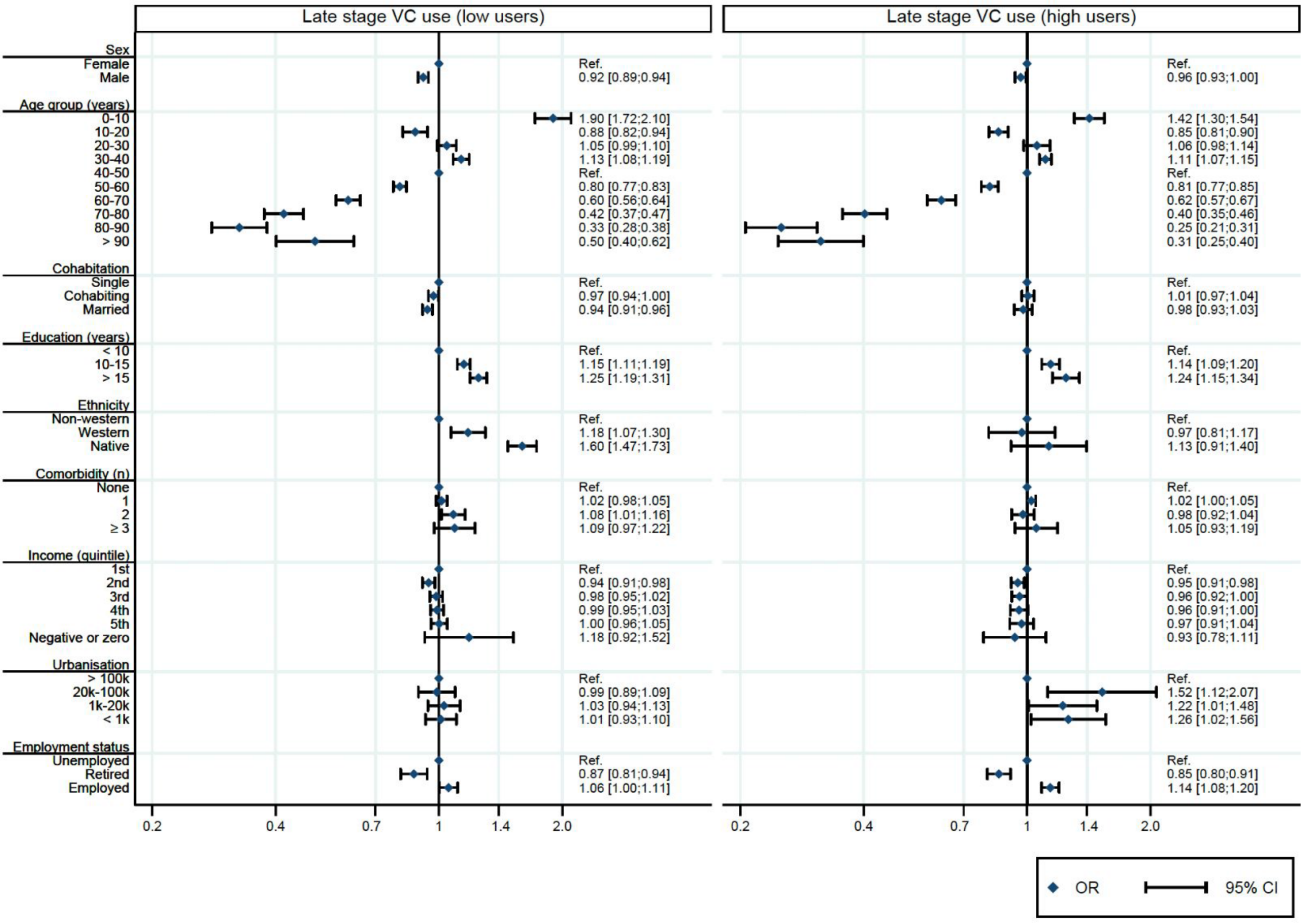
Adjusted^a^ odds ratio (95% confidence interval) for having a video consultation for different patient characteristics when contacting a video-using practice, stratified for low- and high-using practices in the late-stage pandemic. Definition of video-using GP practice based on late-stage pandemic period (14 June 2020–30 November 2021). ^a^Mutually adjusted for variables included in the figure. 95% CI = 95% confidence intervals.OR = odds ratio. VC = video consultation

## Results

### Study population

**Figure 4.**
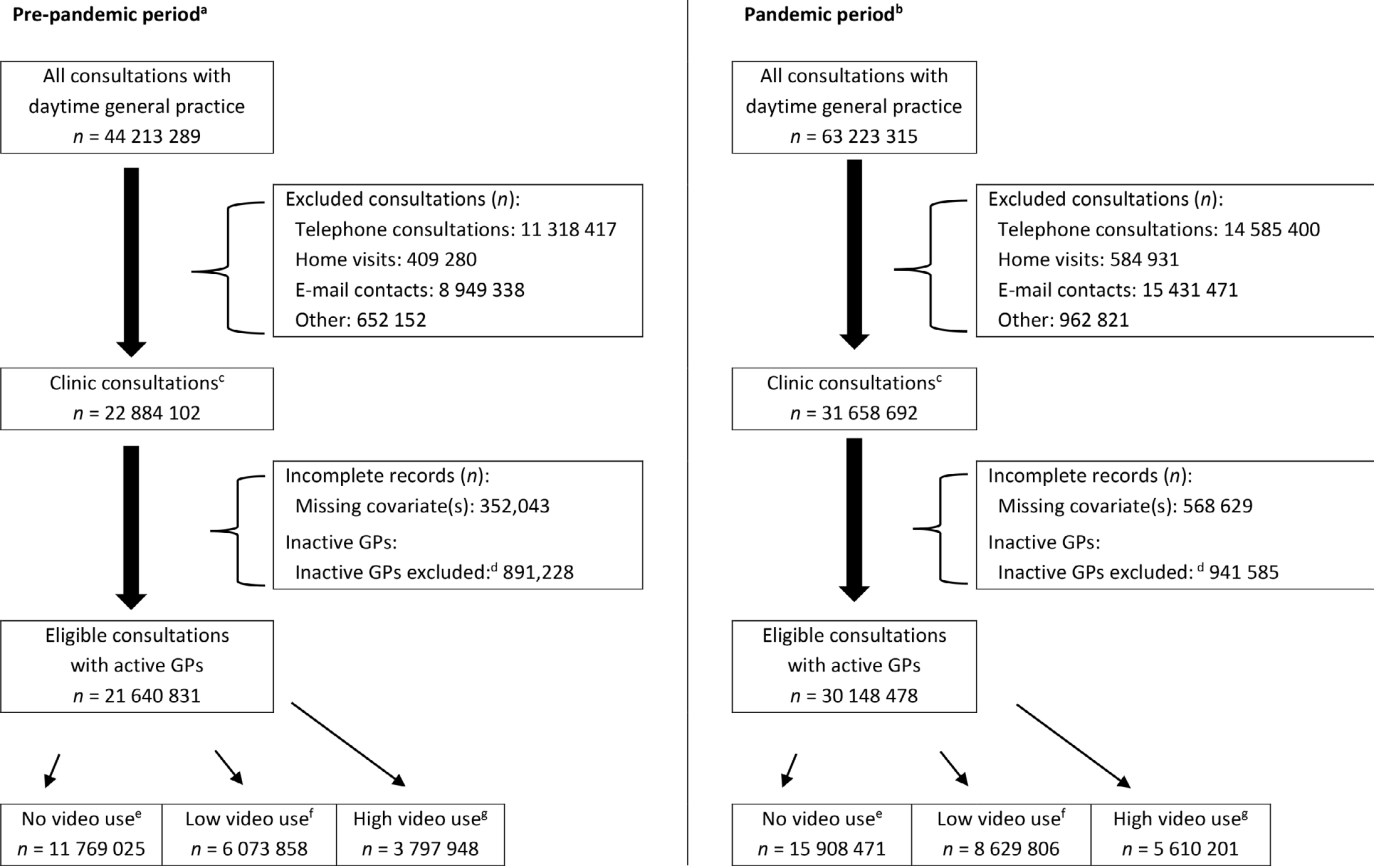
Selection of consultations, stratified for pre-pandemic and pandemic period. ^a^Pre-pandemic period: 1 January 2019–14 March 2020. ^b^Pandemic period: 15 March 2020–30 November 2021. ^c^Clinic consultations included regular (face-to-face) clinic consultations, video consultations, chronic care, and conversational therapy. ^d^<300/1000 clinic consultations during the early stage pandemic period (15 March 2020–13June 2020) AND <700/1000 clinic consultations during the late-stage pandemic period (14 June 2020–30 November 2021). User rate based on the entire pandemic period: ^e^No use: user rate <1%; ^f^Low use: user rate 1–3%; ^g^High use: user rate >3%.

We included 30 148 478 eligible consultations during the study period; 81.4% were registered at practices with no or low video use, and 18.6% were registered at practices with high video use (Figure 4).

### User rate of video consultations

Video was used mostly during the early stage pandemic period, with an average user rate of 8.5% out of all clinic consultations in March 2020, peaking at 11.0% on a single day, before declining to 2.0% in the late-stage pandemic period (Figure 1). Small variations existed between regions (data not shown).

Table 1 compares the pre-pandemic patient population, which received clinic consultations in GP practices with no, low, and high video use in the early and late-stage pandemic periods. The characteristics of the pre-pandemic population did not vary between GP practices' video-user rate between the early and late-stage pandemic periods. Practices with high video use had younger patients in clinic consultations compared with practices with no and low video use. Furthermore, high-use practices had more highly educated patients, patients without comorbidity, patients with higher income levels, and patients in urban areas in their clinic consultations.

### Patient characteristics associated with video use in the early stage and late-stage pandemic period

Figure 2 presents the odds of having a VC when contacting a practice with low or high video use in the early stage pandemic period for different patient characteristics. When contacting a practice with high video use, children aged 0–10 years had significantly higher odds (aOR = 1.50, ref: patients aged 40–50 years) of receiving a VC, compared with the reference category, whereas older patients had lower odds (aOR range = 0.43–0.87). Furthermore, patients with longer education (≥10 years: aOR range = 1.11–1.23, ref:<10 years) and those being employed (aOR = 1.13, ref: unemployed) had significantly higher odds of receiving a VC than the reference groups. Moreover, patients with lower income (aOR range = 0.93–0.94, ref: lowest quintile) and patients living outside big cities (aOR range = 0.81–0.82, ref: urban) had significantly lower odds of receiving a VC than the reference groups. A similar pattern was seen for practices with low video use, although with minor differences. However, in practices with low video use, native Danes and western immigrants had significantly higher odds of receiving a VC than non-western immigrants (aOR range = 1.22–1.51), and patients with ≥2 comorbidities had lower odds than patients without comorbidities (aOR range = 0.78–0.88).

Figure 3 presents the odds of getting a VC in the late-stage pandemic period. When approaching a practice with high video use, children aged 0–10 years and adults aged 30–40 years had significantly higher odds (aOR = 1.42 and 1.11, respectively) of receiving a VC, compared with the reference category (patients aged 40–50 years), whereas adolescents aged 10–20 years and patients aged >50 years had lower odds (aOR range = 0.25–0.85). Furthermore, patients with longer education (≥10 years: aOR range = 1.14–1.24, ref:<10 years) and patients being employed (aOR = 1.14, ref: unemployed) had significantly higher odds of getting a VC than the reference groups.

Additionally, patients with lower income (aOR range = 0.95–0.96, ref: lowest quintile) and patients who had retired (aOR = 0.85) had significantly lower odds of getting a VC than the reference groups. This pattern was mostly similar for practices with low video use. However, native Danes and western immigrants had significantly higher odds of getting a VC (aOR = 1.60 and 1.18, respectively) than non-western immigrants, and patients with two or more comorbidities seemed to have higher odds than patients without comorbidities (aOR range = 1.08–1.09).

## Discussion

### Summary

Video use in daytime general practice peaked at the beginning of the pandemic period. At this time, VCs comprised up to 11.0% of all clinic consultations on a single day. After this peak, it levelled out at around 2.0%. Video-using GP practices provided VCs more often to children and highly educated patients, whereas VCs were less often offered to patients with comorbidities, patients with medium to low income, and patients in suburban and rural areas.

### Strengths and limitations

We used a large dataset, which included all clinic consultations with general practices in Denmark during an extensive period and comprised a wide range of patient characteristics. The results of this study can be generalised to other countries with a similar healthcare system that is free of charge for the patient and based on GP gatekeeping. The data were based on regular coding, which is known to be useful for research purposes,^17^ as completeness is generally obtained owing to the economic incentive for GPs to register their delivered services. Even though this is a strength, some reservations should be noted, in particular owing to the use of rapidly introduced temporary COVID-19 remuneration codes. Moreover, we explored patient characteristics associated with utilising VCs, but the study design did not allow us to assess the clinical and sociocultural aspects of the condition, (digital) health literacy or cognitive considerations,^24^ reluctance to physical contact, lower level of illness, or reduced availability and accessibility of general practice.^15^

### Comparison with existing literature

We found a video-user rate of up to 11.0% for all clinic consultations at the beginning of the early stage pandemic period, which later dropped to around 2.0%. These findings do not unambiguously correspond to the findings reported in other studies of general practice consultations during the COVID-19 pandemic. Murphy *et al* ^20^ showed that 1% of consultations overall occurred by video in the UK, whereas NHS Digital in England revealed that video and e-consultations combined accounted for no more than 0.5% of general practice consultations in December 2021.^21^ In line with our findings, two studies have also shown an uptake of VCs in primary care of up to 2%.^22,23^ GPs and patients may be driven to use video when necessary (for example, in a particular crisis context such as the COVID-19 pandemic), but they seem to prefer usual care routines once the external motivational factor is removed.

Our findings on patient characteristics in relation to having a VC show a disparity between patient groups. This is in line with other studies reporting that the pandemic has exposed and magnified difference in use,^12,25,26^ especially for particular populations. Moreover, register-based population studies showed that remote consultations in general practice were most likely offered to younger, affluent, and educated groups, including employed people and non-immigrants.^27,28^

Eberly *et al* investigated difference in access to remote care in the early phase of the COVID-19 pandemic in the US^29^ and found that older, female, poorer, and immigrant patients were offered video use less often than the general population. Similarly, Rodriguez *et al* found that patients aged >65 years, immigrants, and patients living in areas with limited wi-fi access points were less likely to use VC.^30^ However, this is partly contradicted in another study, which found that the uptake of remote care was highest among older people and patients with the highest expected healthcare needs.^31^

Since, in Denmark, both patient and GP can suggest a VC and mutual agreement is needed, multiple factors could explain the difference in VCs between patient groups. Video was implemented to continue care provision during the COVID-19 pandemic, substituting face-to-face contacts in daytime general practice. Although evidence suggests that VCs offer several advantages,^10,32^ video use decreased rapidly post-pandemic, suggesting a preference for face-to-face contacts in this setting. However, video seems to be considered a valid alternative for particular patient groups, such as those with mental health issues^33^ and those in need of palliative care,^34^ as well as for certain contact types such as chronic care management^35,36^ and specialist consultation.^37^ At acute care outside office hours, the level of video use for triage seems higher,^38^ indicating relevance for acute health problems. As such, a difference in VC between patient groups may not be problematic.

### Implications for research and practice

Several studies on VCs in primary care have shown non-adaptation or very poor uptake,^20–23^ which indicates that most GPs perceived no added value of using VC over existing modalities such as telephone and email.^39^ However, we see that VC use continued in daytime general practice in Denmark during the late-stage pandemic. Thus, future research should study post-pandemic video use and identify video-using practices to explore how video use could work for whom and under which circumstances. Moreover, it should focus on the choice of video (that is, GP or patient-initiated) and the rationales behind this choice, to explore the difference in video use across patient groups.

## Data

The data are not publicly available. All data analysed in the study were de-identified and stored on a secure server at Statistics Denmark. The datasets are available after formal application to Statistics Denmark.
